# Emergence of Vulvovaginal Candidiasis among Lebanese Pregnant Women: Prevalence, Risk Factors, and Species Distribution

**DOI:** 10.1155/2019/5016810

**Published:** 2019-07-28

**Authors:** Nahed Ghaddar, Ali El Roz, Ghassan Ghssein, José-Noel Ibrahim

**Affiliations:** ^1^Faculty of Public Health, Lebanese German University (LGU), Sahel Alma, Lebanon; ^2^Faculty of Public Health II, Lebanese University, Fanar, Lebanon

## Abstract

**Objective:**

* Candida* species colonize the vagina in at least 20% of women, with rates rising to 30% during pregnancy. This study aimed at determining the prevalence and risk factors of vulvovaginal candidiasis (VVC) in pregnant women at 35-37 weeks of gestation. It also aims at finding possible correlations between VVC and vaginal colonization by other agents, such as Group B* Streptococcus *(GBS) and bacterial vaginosis.

**Methodology:**

Over a one-year period, high vaginal swabs were collected from pregnant women during their regular antenatal checkup in different polyclinics in Beirut and South Lebanon. Swabs were examined microscopically, cultured on Sabouraud Dextrose Agar, and* Candida* isolates were identified using Chromatic* Candida* medium and Germ Tube Test.

**Results:**

VVC was detected in 44.8% of samples, with* C. glabrata* (44.4%) and* C. albicans* (43.4%) being the most isolated species. Approximately, half of pregnant women (57.7%) were coinfected with* Candida* and bacterial vaginosis, while 26% of them carried simultaneously* Candida* spp. and GBS. No significant correlation was found between the occurrence of VVC and demographic, clinical, medical, and reproductive health characteristics of pregnant women. In contrast, participants with previous miscarriages and those being hospitalized during the past 12 months were more susceptible to develop vaginal* C. krusei* infection in comparison to other* Candida* species (p=0.0316 and p=0.0042, respectively).

**Conclusion:**

The prevalence of VVC in pregnant women is an increasing trend in our community. Therefore, routine medical examination and regular screening for candidiasis in the antenatal care program is highly recommended to manage the disease and its complications.

## 1. Introduction

Vulvovaginal candidiasis (VVC) is an opportunistic mucosal infection and the second most common vaginal infection affecting women of reproductive age [1]. It affects more than 75% of women at least once in their lifetime, with approximately 50% of them also suffering a single recurrence [1, 2]. The clinical symptoms and manifestations of VVC include cottage cheese-like vaginal discharge, swelling, pruritus, pain, irritation, burning sensation, dyspareunia, and dysuria [1, 3].

Several studies have shown that prevalence of* Candida* among pregnant women is higher than that in nonpregnant women, and it tends to increase with the progression of the pregnancy [4–7]. Some emerging data have also suggested that VVC during pregnancy might be associated with increased risk of complications, such as premature rupture of membranes, preterm labor, chorioamnionitis, and congenital cutaneous candidiasis [8].

Different factors related to physiologic changes, such as decreased cellular immunity, elevated hormone levels, reduced vaginal pH, and increased vaginal glycogen concentration, have been associated with a higher risk of VVC during pregnancy [6, 9, 10].* Candida* colonization is also considered to be a consequence of broad-spectrum antibiotic use and has been reportedly associated with a decreased colonization of* Lactobacillus*, probably resulting from the interference with epithelial binding sites [5]. Indeed, when balance between* Candida*, normal bacterial flora, and immune defense mechanisms is disturbed, colonization is replaced by infection.

According to most reports,* C. albicans* is responsible for the largest number of VVC, contributing to 85-90% of symptomatic episodes of vaginal candidiasis [5]. Non-*albicans Candida* species, including among others* C. glabrata*,* C. tropicalis*, and* C. krusei*, are now emerging as identifiable causes of VVC and differ considerably with regard to epidemiology, virulence, and antifungal drug susceptibility [2, 3, 11].

Invasive candidiasis in neonates is becoming a serious and common cause of late onset sepsis, with mortality rates reaching as high as 25-35% [12]. The incidence of such fungal infections has increased 11-fold over the past 15 years, and* Candida* species are currently considered as the third most frequent organism isolated in late onset sepsis in very low birth weight infants [12].

In this regard, the present study was conducted to determine the prevalence of VVC and other vaginal colonization among Lebanese pregnant women, as well as the frequency of distribution of* C. albicans* and non-*albicans Candida* species in vaginal cultures. It also aims to assess the demographic and clinical profiles of pregnant women and to determine risk factors contributing to the emergence of the disease during pregnancy.

## 2. Materials and Methods

### 2.1. Study Design and Samples Collection

The study was conducted according to the Declaration of Helsinki and approved by the Ethical Committee of Notre Dame University Hospital. A total of 221 pregnant women, in their 35-37 weeks of gestation, presenting at the outpatient clinic of obstetrics and gynecology department of different hospitals and peripheral clinics in Lebanon, were enrolled in this study between September 2016 and August 2017. After obtaining a written and verbal informed consent from all participants, vaginal swabs were collected by the gynecologist from pregnant women during their regular antenatal care checkup using a one cotton-tipped swab with Stuart media (Oxoid, United Kingdom). Samples were then submitted to culture, and a questionnaire was filled by the attending physician for the first 107 participants enrolled in the study to gather relevant information regarding socio-demographic data, clinical symptoms, personal medical history, treatment, and risk factors for vaginal colonization.

### 2.2. Isolation and Identification of* Candida* Species

Vaginal swabs were inoculated on Sabouraud Dextrose Agar (SDA) (Liofilchem, Italy) for fungi isolation and incubated at 37°C for 48h. Direct wet mount examination and Gram staining were performed for all suspected colonies, and isolates were recultured on SDA for 48h at 35°C for purity purposes. Positive* Candida* cultures were further identified to the species level by the Chromatic* Candida* medium (Liofilchem, Italy) that allows the selective isolation and differentiation of* Candida* spp. based on colony color and morphology. The chromatic agar has been well documented in previous studies as for its high sensitivity and specificity for the identification of the most commonly encountered* Candida *spp. [13–15]. After 72h of incubation of Chromatic* Candida* plates at 37°C, green colonies were identified as* C. albicans*, creamy colored colonies were regarded as* C. glabrata*, and* C. krusei* colonies were pink with a whitish border.* C. albicans* isolates were further confirmed by germ tube test.

### 2.3. Isolation and Identification of Bacterial Colonization and Bacterial Vaginosis

Swabs were cultured in various solid selective media, namely 5% blood agar for the isolation of Group B* Streptococci* (GBS), and Mannitol Salt agar (Liofilchem, Italy) for* Staphylococcus aureus* isolation. After 24h incubation at 37°C, colonies were identified routinely by morphological and biochemical tests. Bacterial vaginosis identification was based on the obstetrician clinical assessment or on the detection of clue cells in the direct Gram staining.

### 2.4. Statistical Analysis

Statistical analyses were performed using GraphPad Prism 6 (GraphPad Software, Inc., San Diego, CA, USA). Means and standard deviations for quantitative variables were calculated. The search for factors associated with vaginal candidiasis was performed by Chi square exact test comparing frequencies between groups. Fisher's exact test was used where appropriate. P values less than 0.05 were considered significant.

## 3. Results 

### 3.1. Demographic and Clinical Characteristics of Participants

Based on the questionnaire filled by 107 pregnant women, the participants' age ranged from 20 to 40 years. The majority of studied women (71.9%) had a tertiary education level, while only 8.4% of them were illiterate. A symptomatic VVC was detected in 82% of pregnant women, with the most frequently reported symptoms including abnormal vaginal discharge (67%), itching (22%), and malodor (11%).

### 3.2. Prevalence of VVC and Bacterial Vaginal Colonization

Out of the 221 high vaginal swabs collected during the study period, 99 (44.8%) were found to be positive for* Candida*. The prevalence of* C. albicans* infections was 43.4%, while non-*albicans Candida *strains counted for 56.6% of VVC, the main identified species being* C. glabrata* (44.5%) followed by* C. krusei *observed only in 12.1% of vaginal candidiasis. In 2% of cases, women were infected with* C. albicans* and* C. glabrata* concurrently.

On the other hand, rates of bacterial colonization were, respectively, 11.7% (n=26) for bacterial vaginosis, identified by the presence of vaginal discharge and clue cells visualized under microscope, 20.8% (n=46) for GBS and 5% (n=11) for* Staphylococcus aureus*. The most prevalent coinfection with* Candida* was bacterial vaginosis as 57.7% of pregnant women with bacterial vaginosis carried simultaneously different* Candida* species. However, only 26% of colonized women were coinfected with GBS and* Candida*.

### 3.3. Correlation of Demographic and Clinical Characteristics of Participants with the Occurrence of VVC

The isolation rates of VVC with regard to age, level of education, and type of symptoms are shown in Table 1. Women aged between 26 and 30 years and those with primary education level had the highest frequencies of* Candida*-positive cultures, but without any significant association between all these factors and the occurrence of VVC (p = 0.4789 and p=0.2266, respectively) (Table 1). Moreover, no significant correlation was found between the isolation frequencies of* Candida*-positive cultures and the presence of clinical symptoms, namely vaginal discharge, itching, and malodor (p=0.5303, p=0.1826, and p=0.3697, respectively) (Table 1).

### 3.4. Correlation of Demographic and Clinical Characteristics of Participants with* Candida* Species Distribution

Statistical analyses of demographic data of patients showed no significant difference in* Candida* species distribution with regard to age (p=0.7755) and level of education (p=0.1994) (Table 2). Moreover, correlation of vulvovaginal symptoms with* Candida* species distribution showed that vaginal discharge was more frequent in* C. albicans* (44.4%) rather than non-*albicans *vaginal candidiasis. As for itching, it was exclusively found in* C. albicans *infections, while malodor rates were comparable regardless of the species isolated (Table 2). While vaginal discharge and malodor seem not to be related to* Candida* species distribution (p=0.4293 and p=0.6276, respectively), the presence of itching in pregnant women was found to be significantly associated with a higher occurrence of* C. albicans* infections (p=0.0317) (Table 2).

### 3.5. Correlation of Medical and Reproductive Health Characteristics of Participants with VVC Prevalence and* Candida* Species Distribution

The prevalence of VVC and the distribution of* Candida* species were also assessed in relation to patient's medical history, as well as to previous and current treatment and reproductive health characteristics. As shown in Table 3, there was no significant difference in the frequencies of* Candida* infection with regard to all variables investigated in the present study, thus suggesting that none of these variables can be considered as a risk factor for vaginal candidiasis in Lebanese pregnant women.

Interestingly, women with previous miscarriages and those being hospitalized during the past 12 months showed a significant differential distribution in* Candida* species, with an increased susceptibility to develop vaginal* C. krusei* infection in comparison to other* Candida* species (p=0.0316 and p=0.0042, respectively) (Table 4).

## 4. Discussion

VVC represents one of the most frequent infections of the genital tract affecting millions of women each year [2]. Several studies evaluating the prevalence of candidiasis among pregnant women showed that the distribution of isolated* Candida* species varies between countries, and it is greatly depending on several risk factors such as age, hygienic habits, and disease history [2, 6, 10, 16]. To the best of our knowledge, this is the first study investigating the prevalence of vaginal candidiasis among pregnant women in Lebanon. Our results showed a rate of 44.8% of VVC among the studied population which is clearly higher than that observed by Ramia* et al.* in 2012 on a sample of 441 Lebanese nonpregnant women [17], thus highlighting the higher occurrence of VVC during pregnancy. Data obtained were on the other hand comparable to frequencies reported by studies conducted in Libya, Tanzania, Kenya, and Turkey (43.8%, 42.9%, 42.7%, and 37.4%, respectively) [6, 18, 19], but higher than that observed in Kuwait (13%) [2].

The high rate of infection observed in this study can be attributed to the fact that* Candida* infections tend to increase throughout pregnancy due to high levels of placental estrogens, progesterone, and corticosteroids that can reduce the vaginal defense mechanisms and facilitate the growth of yeasts [20, 21]. Moreover, pregnant women are usually more prone to infections because of the weakened immune system and the prolonged misuse of antibiotics during pregnancy [19, 22, 23].

As reported by previous studies, the majority of participants (94%) harbored only one* Candida* species, whereas only 6% of pregnant women were coinfected with more than two yeast isolates [2, 3, 24].* C. glabrata* was the most frequently isolated species (44.5%) followed by* C. albicans* (43.4%) and* C. krusei* (12.1%). The higher percentage of occurrence of VVC attributed to non-*albicans Candida* species (56.6%) compared to that related to* C. albicans* species is consistent with data reported in recent studies [25]. Indeed, a remarkable increase in the incidence of isolation rate of non-*albicans Candida* species has been recently observed in several Asian and African countries, with* C. glabrata* regarded as the most common associated species with VVC [2, 3, 16, 20, 24–26]. These non-*albicans Candida* species are comparatively nonpathogenic, but eventually they are elected, and begin emerging frequently due to the extensive misuse of over the counter antifungals, the use of single-dose oral and topical azoles treatments, and the long-standing protection treatments of oral azoles [25].

The distribution of* Candida* infection frequencies with regard to age and level of education showed that women aged between 26 and 30 years and those with primary education had the highest rates of infection (Table 1). These results may be related to the high sexual activity of participants belonging to this group as well as to the fact that these women are more likely to indiscriminate drug and contraceptives usage to prevent pregnancy [18]. In agreement with findings from other groups [11, 18, 27],* C. albicans* was the predominant isolate from women of childbearing age (Table 2). Indeed, the high levels of estrogen in the latter group induce the formation and deposition of glycogen in the vagina, thus providing a favorable environment for the growth of* C. albicans* [28]. As for results regarding the level of education, they may be related to the lack of knowledge of women with primary education in preventing and reducing risk of candidiasis.

The most common symptoms observed in Lebanese pregnant women with VVC were in accordance with findings in the literature and included mainly vaginal discharge, itching, and malodor that can interfere with the normal daily life of women [1]. Analysis of the distribution of these symptoms among different* Candida* species revealed that vaginal discharge and itching are more frequent in* C. albicans* in comparison to other* Candida* species, as it would be presumably due to its virulence [9].

The increased risk for VVC during pregnancy may be related to several factors, such as sociodemographic and clinical profile of patients, as well as personal medical history, reproductive health characteristics, and previous and current treatment. In this regard, we investigated the possibility of an association between all these factors and susceptibility to VVC. As shown in Tables 1 and 3, none of the factors investigated was significantly associated with the occurrence of VVC, thus suggesting that all of them cannot be considered as major determinants for* Candida* infection in Lebanon. Previous reports have revealed that multigravida women more often suffer from VVC due to longer sexual histories and more pregnancies, hence making them more prone to developing VVC [6]. However, in the present study the number of children did not correlate with the occurrence of* Candida* infection, which may be related to the limited sampling number. Findings related to patient's medical history and use of antibiotics were similar to some reports [4, 29, 30] but contradictory to others [9, 22, 31] and may be attributed to differences in study designs, as well as geographic determinants, dietary and hygiene habits, and sociocultural traits of participants.

Interestingly, our results showed a significant association between the presence of previous miscarriages and hospitalization with* Candida* species distribution. Women with previous miscarriages and those being hospitalized during the past 12 months were more likely to develop* C. krusei* infection in comparison to other* Candida* species (p=0.0316 and p=0.0042; Table 3).* C. krusei* has been described as a causative agent of disseminated fungal infections in susceptible patients and has been associated with a statistically significant increased risk of spontaneous abortion in pregnant women treated with antifungals [32].

## 5. Conclusion

The high frequency of* Candida* recovered among Lebanese pregnant women represents a major public health concern. Additional studies on a larger sample and evaluating other potential factors related to VVC are of great importance to prevent and reduce risk of* Candida* infections among Lebanese pregnant women. Moreover, the role of non-*albicans Candida* in recurrent VVC warrants further investigation, due to the increased frequency of resistant non-*albicans Candida* species isolated from vaginal samples. Routine medical examination, adequate antenatal services, and regular screening for candidiasis with antifungal susceptibility testing are highly recommended for a better monitoring of the disease and its burden among Lebanese pregnant women.

## Figures and Tables

**Table 1:** Frequency of *Candida* infections with regard to socio-demographic and clinical characteristics of patients.

Variables	Negative N (%)	Positive N (%)	*P* value
Age group			
20 -25	7 (87.5)	1 (12.5)	
26 – 30	27 (65.9)	14 (34.1)	0.4789
31 - 40	40 (69)	18 (31)	

Level of education			
None	6 (66.6)	3 (33.3)	
Primary	5 (62.5)	3 (37.5)	0.2266
Secondary	9 (69.9)	4 (30.7)	
Tertiary	54 (70.2)	23 (29.8)	

Clinical symptoms			
*Discharge*			
Yes	34 (65.4)	18 (34.6)	0.5303
No	40 (72.8)	15 (27.2)	
*Itching*			
Yes	6 (50)	6 (50)	0.1826
No	68 (71.6)	27 (28.4)	
*Malodor*			
Yes	3 (50)	3 (50)	0.3697
No	71 (70.3)	30 (29.7)	

N: number of individuals; Chi square test was performed to generate p values for differences in *Candida *infection frequencies with regard to age, level of education, and clinical symptoms. Fisher's exact test was used where appropriate.

**Table 2:** * Candida* spp. distribution with regard to patients' demographic and clinical symptoms.

Variables	*C. albicans* N (%)	*C. glabrata* N (%)	*C. krusei* N (%)	*P* value
Age group				
20 -25	1 (100)	0 (0)	0 (0)	0.7755
26 – 30	6 (42.8)	5 (35.7)	3 (21.5)	
31 - 40	10 (55.5)	6 (33.3)	2 (11.2)	

Level of education				
None	2 (66.7)	1 (33.3)	0 (0)	0.1994
Primary	0 (0)	1 (33.3)	2 (66.7)	
Secondary	2 (50)	2 (50)	0 (0)	
Tertiary	13 (56.5)	7 (30.4)	3 (13.1)	

Clinical Symptoms				
*Discharge*				
Yes	8 (44.5)	6 (33.3)	4 (22.2)	0.4293
No	9 (60)	5 (33.3)	1 (6.7)	
*Itching*				
Yes	6 (100)	0 (0)	0 (0)	0.0317
No	11 (40.7)	11 (40.7)	5 (18.6)	
*Malodor*				
Yes	1 (33.3)	1 (33.3)	1 (33.3)	0.6276
No	16 (53.3)	10 (33.3)	4 (13.4)	

N: number of individuals; Chi square test was performed to generate p values for differences in *Candida* species distribution with regard to age, level of education, and clinical symptoms.

**Table 3:** Frequency of *Candida* infections with regard to reproductive health characteristics, patient's medical history, and previous and current treatment.

Variables	Negative N (%)	Positive N (%)	*P* value
Previous Miscarriage			
Yes	22 (68.8)	10 (31.2)	1
No	52 (69.4)	23 (30.6)	

Number of Children			
0	40 (71.5)	16 (28.5)	0.8677
1	26 (66.7)	13 (33.3)	
*⩾* 2	8 (66.7)	4 (33.3)	

Clinical History			
None	37 (74)	13 (26)	0.2435
Diabetes	13 (50)	13 (50)	
Gestational Diabetes	4 (57.2)	3 (42.8)	
Anemia	12 (57.2)	9 (42.8)	
Urinary Tract Infection	0 (0)	1 (100)	
Recurrent vaginitis	1 (50)	1 (50)	

Vaginitis			
Yes	12 (57.2)	9 (42.8)	0.1970
No	62 (72.1)	24 (27.9)	
Vaginosis			
Yes	8 (72.8)	3 (27.2)	1
No	66 (68.8)	30 (31.2)	

Treatment			
*Antibiotic*			
Yes	2 (33.3)	4 (66.7)	0.0715
No	72 (71.3)	29 (28.7)	
*Antiseptic*			
Yes	46 (69.7)	20 (30.3)	1
No	28 (68.3)	13 (31.7)	
*Antifungal*			
Yes	4 (57.2)	3 (42.8)	0.6738
No	70 (70)	30 (30)	

Previous GBS isolation			
Yes	4 (80)	1 (20)	0.6646
No	70 (68.7)	32 (31.3)	

Antibiotic over last 12 months			
Yes	12 (63.2)	7 (36.8)	0.5877
No	62 (70.5)	26 (29.5)	

Hospitalization last 12 months			
Yes	6 (54.5)	5 (45.5)	0.3086
No	68 (70.9)	28 (29.1)	

N: number of individuals; Chi square test was performed to generate p values for differences in *Candida *infection frequencies with regard to reproductive health characteristics, patient's medical history, and previous and current treatment; Fisher's exact test was used where appropriate.

**Table 4:** * Candida* spp. distribution with regard to reproductive health characteristics, patient's medical history, and previous and current treatment.

Variables	*C. albicans* N (%)	*C. glabrata* N (%)	*C. krusei* N (%)	*P* value
Previous Miscarriage				
Yes	4 (40)	2 (20)	4 (40)	0.0316
No	14 (60.8)	8 (34.8)	1 (4.4)	

Number of Children				
0	8 (50)	7 (43.8)	1 (6.2)	0.3556
1	7 (53.8)	3 (23.1)	3 (23.1)	
*⩾* 2	3 (75)	0 (0)	1 (25)	

Medical History				
None	9 (69.2)	3 (23.1)	1 (7.7)	0.6644
Diabetes	6 (46.1)	4 (30.7)	3 (23.2)	
Gestational Diabetes	3 (100)	0 (0)	0 (0)	
Anemia	3 (33.3)	4 (44.5)	2 (22.2)	
Urinary Tract Infection	1 (100)	0 (0)	0 (0)	
Recurrent vaginitis	1 (100)	0 (0)	0 (0)	

Vaginitis				
Yes	5 (55.6)	2 (22.2)	2 (22.2)	0.7127
No	13 (54.1)	8 (33.3)	3 (12.6)	
Vaginosis				
Yes	2 (66.7)	1 (33.3)	0 (0)	0.7412
No	16 (53.3)	9 (30)	5 (16.7)	

Treatment				
*Antibiotic*				
Yes	3 (75)	1 (25)	0 (0)	0.5295
No	14 (48.2)	10 (34.4)	5 (17.4)	
*Antiseptic*				
Yes	11 (55)	5 (25)	4 (20)	0.3744
No	6 (46.1)	6 (46.1)	1 (7.8)	
*Antifungal*				
Yes	3 (100)	0 (0)	0 (0)	0.2116
No	14 (46.6)	11 (36.6)	5 (16.8)	

Previous GBS isolation				
Yes	1 (100)	0 (0)	0 (0)	0.6115
No	16 (50)	11 (34.3)	5 (15.7)	

Antibiotic over last 12 months				
Yes	4 (57.1)	3 (42.9)	0 (0)	0.4399
No	13 (50)	8 (30.7)	5 (19.3)	

Hospitalization last 12 months				
Yes	0 (0)	2 (40)	3 (60)	0.0042
No	17 (60.7)	9 (32.1)	2 (7.2)	

N: number of individuals; Chi square test was performed to generate p values for differences in *Candida* species distribution with regard to reproductive health characteristics, patient's medical history, and previous and current treatment.

## Data Availability

The data in the tables used to support the findings of this study are included within the article.
